# Relationship between the volume and type of appointments in general practice and patient experience: an observational study in England

**DOI:** 10.3399/BJGP.2024.0276

**Published:** 2025-03-25

**Authors:** Patrick Burch, William Whittaker, Yiu-Shing Lau

**Affiliations:** Centre for Primary Care, University of Manchester, Manchester.; Public Health, Policy & Systems, University of Liverpool, Liverpool.; Health Organisation, Policy and Economics Group, University of Manchester, Manchester.

**Keywords:** delivery of health care, general practice, patient satisfaction, primary health care

## Abstract

**Background:**

Patient satisfaction is a significant dimension of quality in general practice and has notably declined post-COVID. Understanding the dynamics between practice activities, practice characteristics, and patient experience is vital for improving care quality.

**Aim:**

To investigate the relationship between the volume, modality (telephone or face to face), and practitioner type of general practice appointments and patient experience.

**Design and setting:**

This was an observational study of general practices in England.

**Method:**

Data from the GP Patient Survey (GPPS) were merged with NHS England’s practice-level appointment data, covering August 2022 to March 2023. Ordinary least squares regressions were estimated of patient satisfaction with access, general satisfaction, preference for a specific GP, and support for managing long-term conditions (dependent variables) against appointment volume, modality (telephone or face to face), and practitioner type.

**Results:**

Analysis of 5278 practices showed that a higher volume of appointments, especially face to face with GPs, was significantly (*P*<0.001) associated with increased patient satisfaction. Practices having a greater proportion of same-day appointments was significantly correlated with lower patient satisfaction.

**Conclusion:**

Patient satisfaction and ability to have health needs met is associated with face-to-face access to GPs as well as the total volume of appointments available. The results suggest that patients’ perceptions of access involve more than immediate availability of appointments or that patients may struggle to get appointments at practices offering more same-day appointments. Initiatives to improve access to, and satisfaction with, general practice should prioritise expanding face-to-face GP appointments.

## Introduction

Patient experience of general practice is recognised as one independent marker of quality.^1^ Prior to the COVID pandemic patient satisfaction with and experience of general practice had been declining and this is even more pronounced following the pandemic.^2^ The reasons for this decline are complex and not fully understood. Practice funding, increased patient demand, waiting times, changing patient expectations, workforce issues, and an increase in remote consultations have all been hypothesised as contributing.^3^^–^^5^

One way to understand the factors affecting patient experience is to examine the association between individual practice characteristics and patient experience. The largest reliable source of patient experience data in England is the GP Patient Survey (GPPS).^6^ Existing research has used the GPPS to examine the link between general practice patient experience and, for example, emergency department use, availability of extended access, type of health professionals employed, practice size, and use of out-of-hours care.^7^^–^^12^ Several of these factors are specifically trying to understand the impact of expansions in capacity or are measures that will proxy for relative demand pressures. Salant *et al* used GPPS data to show a positive link between practice funding, staffing, and patient experience.^13^ However, until recently there have been no practice-level appointment data available, meaning assessment of the relationship between the provision of routine/core primary care appointments and patient experience has not been possible. In October 2022 NHS England made practice-level appointment data publicly available for all practices in England.^14^

The release of practice-level appointment data was initially criticised by some GPs who felt it may not accurately reflect the workload of practices.^15^ NHS England acknowledged this and stated that data were to be treated as experimental.^14^ The data do have limitations:

- the dataset records how long after booking the patient had their appointment; it does not record how long a patient waited to book an appointment; and
- appointments are classified as with a GP or with a non-GP healthcare professional. The type of non-GP staff carrying out patient appointments are not recorded.

However, while acknowledging these caveats, the data do provide a nationwide measure of practice appointment volume and types, and studies have been carried out using the dataset.^16^

In this article, appointment data were combined with patient-reported indicators from the GPPS to examine, at a practice level, whether there is an association between appointment volume, modality (telephone or face to face), and practitioner type (medical or non-medical) and:

- access (patient satisfaction with appointment times);
- satisfaction (overall satisfaction with general practice);
- continuity (ability to consult with a preferred GP); and
- unmet health needs.

**Table:** How this fits in

It was not previously known, to the authors’ knowledge, how the volume and type of appointments offered by GP practices correlate with patient-reported satisfaction. This research provides evidence that higher volumes of face-to-face appointments, particularly with GPs, is associated with enhanced patient satisfaction, better continuity, and a reduction in unmet health need. The analysis found that more on-the-day appointments were negatively associated with patient satisfaction. These findings suggest that initiatives to improve patient satisfaction in general practice should prioritise increasing the availability of face-to-face GP consultations.

Two questions were chosen to represent important outcomes and indicators of quality of primary care (overall satisfaction and unmet health needs). The other two questions (access and continuity) represent important process measures that are hypothesised to be related to appointment volume, modality, and practitioner type.^17^ All four questions have been used in previous studies investigating the links between appointment provision, morbidity, and patient-reported outcomes.^10^^,^^18^ Patient satisfaction with appointment times is a marker that has been used by NHS England to justify the implementation of access improvement projects.^19^ Improving continuity of care is a priority for NHS England.^20^

The aim of the study was to help inform whether quantity, modality, and/or practitioner type of routine primary care appointments matter to patient experience and, as such, whether initiatives to improve access are better focused on expanding routine general practice appointments (and particular types of appointments).

## Method

Eight months of Appointments in General Practice data were obtained from NHS England, covering the time period from August 2022 until March 2023.^21^ The earliest available data from this dataset were August 2022. The practice-level data contain a total of nine indicators:

- total appointments per registered patient (total appointments; and appointments with a GP);
- appointment time between booking appointment and appointment taking place (percentage: same day; same and next day; within 1 week); and
- appointment mode (proportion of appointments: face to face; telephone; face-to-face appointments with GP; and telephone appointments with a GP).

Data supplier information was also contained in the data. Appointments where patients did not attend, or with unknown status, were included in the data and analysis.

Measures of patient experience were obtained from the 2023 version of the practice-level GPPS data.^22^ The survey was conducted between January and March 2023. This wave of the survey was chosen to, as best possible, align with the practice-level appointment data. As discussed in a previous paper, answers to the GPPS are likely to reflect an accumulated experience with general practice over time, with several questions specifically asking patients to consider their experience over the previous months.^10^ Four measures of patient experience were selected from the data.

- Access: ‘How satisfied are you with the general practice appointment times that are available to you?’ The variable was the percentage for each practice answering: ‘Very satisfied’ or ‘Fairly satisfied’.
- Overall satisfaction: ‘Overall, how would you describe your experience of your GP practice?’ The variable was the percentage for each practice answering: ‘Very good’ or ‘Fairly good’.
- Continuity: ‘How often do you see or speak to your preferred GP when you would like to?’ This was asked to those patients with a preferred GP. The variable was the percentage for each practice answering: ‘Always or almost always’ or ‘A lot of the time’.
- Unmet health needs: ‘In the last 12 months, have you had enough support from local services or organisations to help you manage your condition (or conditions)?’ This was asked to patients with ≥1 long-term conditions. The variable was the percentage of patients answering ‘Yes, definitely’ divided by all those who responded ‘Yes, definitely’ or ‘No’.

The practice-level responder characteristics of age, gender, ethnicity, employment, deprivation, and rurality were also obtained from the survey. Weighted responses from the GPPS were used to ensure representativeness of each practice’s patient population.

General practice workforce data were obtained for additional supply side measures.^23^ The number of full-time equivalent workforce (GP, nurse, direct patient care staff, and administration (admin) staff) per 1000 registered patients was extracted. These measures enable adjustment for workforce differences; in their absence the estimated associations between appointments and patient experience may pick up the effects of workforce variations.

Appointment data, GPPS data, and GP workforce data were linked together at the practice level. To assess associations between patient experience and appointments in general practice ordinary least squares regressions were estimated of each patient experience measure against appointment volume, mode, and practitioner type. Each model controlled for GP practice responder characteristics, workforce characteristics, and GP data supplier information. All models were weighted by general practice-registered patients, and all analysis was performed using Stata 17MP4.

Sensitivity analyses included primary care networks (PCN) and integrated care systems (ICS) fixed-effects in separate models for robustness.

## Results

**Figure 1.**
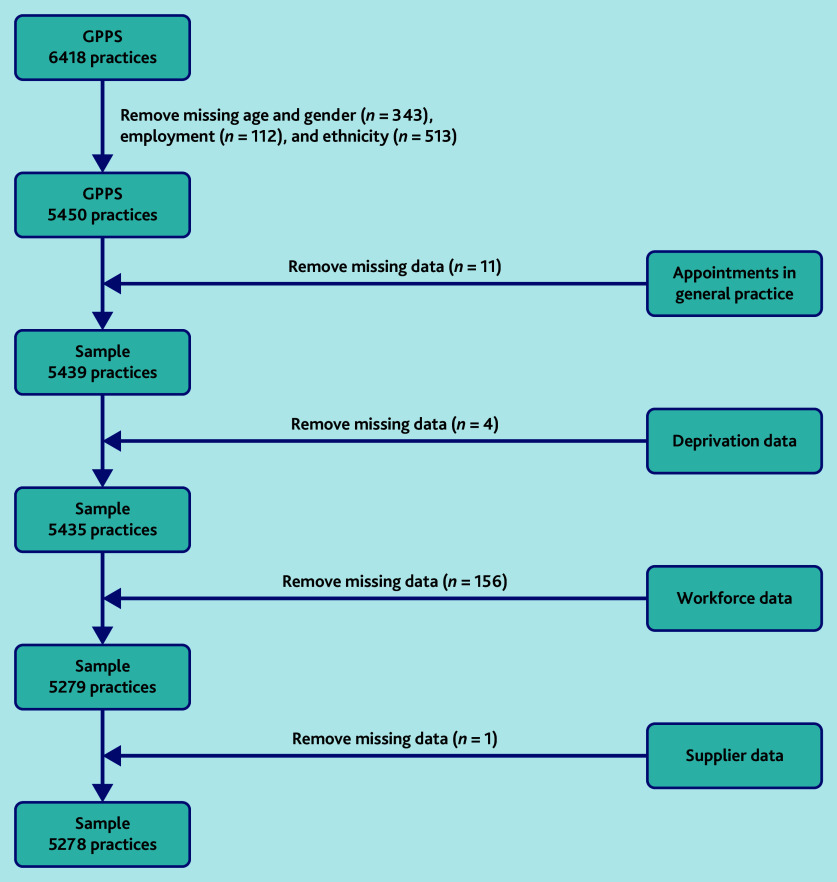
Data inclusion flow chart. GPPS = GP Patient Survey.

As illustrated in Figure 1, 5278 practices had appointment data, GPPS data, workforce, Index of Multiple Deprivation, and rurality data (82.2% of all practices, *N* = 6418), although there were missing data for mode of appointment (ranging from 0.2% [13/5278] to 1.8% [96/5278]). Practices with <1000 registered patients were excluded*.* On average, practices provided 481 appointments per 1000 patients per month, comprised of 230 GP appointments and 251 other appointments (Table 1). In total, a mean of 42.6% of appointments were same day and a mean of 69.9% of appointments were for appointments within a week. Other mean values were: 69.5% of appointments were face to face (27.2% telephone), and 29.6% of appointments were face to face with a GP (18.4% were GP telephone appointments).

**Table 1.** Summary statistics

**Variable**	***N***	**Mean**	**SD**	**p5^a^**	**p50^a^**	**p95^a^**
**Appointments**						
Monthly appointments per 1000 list	5278	480.70	127.57	309.00	465.66	698.10
Monthly GP appointments per 1000 list	5278	229.63	76.90	118.43	223.00	357.13

**Time between booking and appointment, %**						
Same-day appointments	5278	42.63	10.04	26.84	42.22	59.77
0–1 days	5278	50.60	10.18	34.85	50.22	67.90
0–7 days	5278	69.89	10.00	53.39	69.49	86.66

**Appointment mode, %**						
Face to face	5265	69.48	17.06	41.92	70.06	96.19
On telephone	5224	27.21	16.67	1.43	26.84	55.43
GP face to face of overall	5260	29.59	13.66	10.37	27.67	54.62
GP telephone of overall	5182	18.44	12.94	0.53	17.06	41.57

**GPPS outcomes**						
Satisfied with appointment time	5278	52.22	14.17	29.26	51.93	76.08
Overall satisfaction	5278	44.72	13.87	23.26	43.83	68.87
Appointment with preferred GP	5267	32.64	16.22	9.55	30.42	63.59
Enough support for managing health condition(s)	5278	45.01	15.92	19.57	44.46	72.32

**Practice patient characteristics Age groups, years^b^**						
25–34	5278	16.06	7.55	5.73	15.04	29.12
35–44	5278	17.53	6.25	8.10	17.01	28.58
45–54	5278	17.32	4.41	10.38	17.16	24.87
55–64	5278	16.79	4.41	9.28	16.93	23.83
65–74	5278	12.24	4.46	4.95	12.19	19.60
75–84	5278	8.25	3.89	2.15	8.17	14.71
>85	5278	2.89	1.68	0.63	2.70	5.92

**Gender^b^**						
Female	5278	51.79	5.82	42.44	51.80	61.32
Other	5278	1.76	2.11	0	1.09	5.90

**Broad ethnic group^b^**						
Mixed	5278	2.06	2.32	0	1.35	6.58
Asian	5278	10.23	14.67	0	4.85	41.17
Black	5278	4.37	6.9	0	1.59	19.02
Other	5278	2.41	3.5	0	1.10	9.14

**Employment status^b^**						
Part-time	5278	12.65	4.03	6.49	12.33	19.69
Full-time education	5278	4.45	5.78	0	3.45	10.63
Unemployed	5278	4.20	3.70	0	3.29	11.66
Permanently sick	5278	4.58	3.25	0.69	3.92	10.89
Retired	5278	21.03	8.98	6.15	21.39	35.40
Other	5278	7.36	3.67	2.45	6.73	14.15

**Income deprivation^b^**						
Quantile 2	5278	19.18	14.88	0.71	16.72	47.94
Quantile 3	5278	20.34	13.65	2.34	18.23	45.16
Quantile 4	5278	20.39	15.31	0.19	18.07	48.46
Most income deprived	5278	19.95	23.14	0	10.86	70.41

**Rural**	5278	17.47	31.06	0	1.54	98.99

**Practice staff FTE per 1000 list**						
Total number of GPs	5278	0.57	0.25	0.22	0.55	1.01
Total number of nurses	5278	0.27	0.15	0.07	0.24	0.53

**Total number of direct patient care staff**	5278	0.25	0.22	0	0.19	0.70

**Total number of admin staff**	5278	1.16	0.38	0.62	1.14	1.79

**Supplier, practice *N* (%)**	5278					
EMIS	3130 (59.30)					
Other	57 (1.08)					
TPP	2091 (39.62)					

a *Percentiles of the distribution: p5 (5th percentile), p50 (median), and p95 (95th percentile).*

b *Base categories: age groups (16–24 years), gender (male), broad ethnic group (White), employment status (full-time employed), and income deprivation (least income deprived). FTE = full-time equivalent.* N *= number of GP practices. GPPS = GP Patient Survey. SD = standard deviation.*

For the 5278 practices with appointments data, the corresponding GPPS data highlight the low levels of satisfaction among patients. As shown in Table 1, the mean practice-level satisfaction of patients with appointment times was 52.2%. A practice mean of 44.7% of patients reported overall satisfaction with the practice. Continuity of care also appeared to be low with a practice mean of 32.6% of patients being able to see their preferred GP. A practice mean of 45.0% of patients reported having enough support to manage long-term conditions (55.0% therefore reported unmet needs). Missing data were present for the ability to see their preferred GP (0.2%, 11/5278).

Results from the ordinary least squares regressions are provided in Table 2. Volume of appointments was positively associated with satisfaction with appointment times, overall satisfaction, the ability to see a preferred GP, and with reporting of enough care to support management of health conditions. The effects are greater for GP appointment volume.

**Table 2.** Regression output for patient experience and general practice appointments^a^

**Outcomes**	**Satisfied with appointment times**		**Overall satisfaction**		**Proportion seeing preferred GP**		**Enough care to manage conditions**	

	**Coef.**	**95% CI**	**Coef.**	**95% CI**	**Coef.**	**95% CI**	**Coef.**	**95% CI**
**Volume per registered GP patients**								

Monthly appointments per 1000 registered patients	0.02^d^	(0.01 to 0.02)	0.01^d^	(0.01 to 0.02)	0.01^c^	(0.00 to 0.01)	0.01^d^	(0.01 to 0.02)
Monthly appointments per 1000 registered patients seen by GP	0.03^d^	(0.03 to 0.04)	0.03^d^	(0.02 to 0.04)	0.03^d^	(0.03 to 0.04)	0.02^d^	(0.02 to 0.03)

**Percentage**								
Same-day appointment	−0.05^b^	(−0.09 to −0.01)	−0.06^c^	(−0.11 to −0.02)	−0.23^d^	(−0.28 to −0.18)	−0.08^d^	(−0.13 to −0.04)
0–1 days	−0.04^b^	(−0.08 to −0.001)	−0.06^c^	(−0.10 to −0.02)	−0.22^d^	(−0.26 to −0.17)	−0.10^d^	(−0.14 to −0.05)
0–7 days	0.05^b^	(0.01 to 0.10)	0.04	(−0.01 to 0.08)	−0.08^c^	(−0.13 to −0.03)	−0.08^d^	(−0.13 to −0.04)

**Percentage appointment mode**								
Face to face	0.08^d^	(0.05 to 0.10)	0.10^d^	(0.07 to 0.12)	0.09^d^	(0.06 to 0.12)	0.06^d^	(0.04 to 0.09)
Telephone	−0.07^d^	(−0.10 to −0.05)	−0.09^d^	(−0.12 to −0.07)	−0.09^d^	(−0.12 to −0.06)	−0.07^d^	(−0.10 to −0.04)
GP face to face	0.15^d^	(0.12 to 0.18)	0.17^d^	(0.14 to 0.20)	0.20^d^	(0.16 to 0.24)	0.10^d^	(0.07 to 0.13)
GP telephone	−0.07^d^	(−0.10 to −0.03)	−0.08^d^	(−0.12 to −0.05)	−0.03	(−0.07 to 0.01)	−0.05^c^	(−0.08 to −0.01)

a *Estimates are from separate linear regressions of the outcome against the appointment measure (full sets of estimates are provided in Supplementary Tables S1–S9).*

b P*<0.001.*

c P*<0.01.*

d P*<0.05. CI = confidence interval. Coef. = coefficient.*

A greater percentage of appointments being the same day was associated with smaller rates of satisfaction with appointments, overall satisfaction, the ability to see a preferred GP, and with reporting of enough support for those with health condition(s).

The mode of appointment appears to have implications for satisfaction, with face-to-face appointments positively associated with satisfaction, the ability to see a preferred GP, and with reporting of support for those with health condition(s). In contrast, a greater proportion of appointments being delivered by telephone was negatively associated with all measures of patient experience. Looking at the mode of GP-specific appointments, these findings are echoed, albeit the effects are greater for face-to-face appointments and smaller for telephone appointments.

Satisfaction appears greater for those of older age, mixed, Black and other ethnic groups, those in education, in rural locations, and those who are less deprived (Supplementary Tables S1–S4). Asian ethnic groups had lower levels of feeling they had enough support for health condition(s) (Supplementary Table S4). Those not employed and claiming sickness benefits reported being less supported with health condition(s) (Supplementary Table S4).

The volume of GPs per 1000 registered patients is positively associated with all measures of satisfaction (Supplementary Tables S1–S4). Although nurse and direct patient contact (DPC) staff are not associated with satisfaction with appointment times or overall satisfaction, they are negatively associated with ability to see the preferred GP (Supplementary Table S3). DPC staff were also associated with poorer reporting of support with health condition(s) (Supplementary Table S4).

Results were robust to whether ICS or PCN fixed-effects were accounted for (Supplementary Tables S10 and S11).

## Discussion

### Summary

This study used practice-level appointment data to assess the association of volume, mode, and practitioner type of appointments available with patient-reported experience. The study found that greater volumes of appointments, greater proportions of face-to-face appointments, greater proportions of appointments with GPs, and a lower proportion of appointments within 24 h of booking were associated with greater patient satisfaction with access, overall satisfaction, continuity, and fewer unmet health needs.

### Strengths and limitations

To the authors’ knowledge, this is the first study in the UK that has used national practice-level appointment data to understand the relationship between general practice appointments and patient-reported outcomes. The design of the study means the authors cannot know if the association between appointment characteristics and patient experience and satisfaction is casual. It is possible that practices with poorer patient outcomes have tried to make care more accessible by increasing the amount of non-GP staff and having a greater proportion of telephone appointments. As data accrue this will provide opportunities for stronger identification of any causal relationship. Examination of appointment and GPPS data over ≥2 years will enable an examination of whether declining access and overall satisfaction scores precede an increase in non-face-to-face and non-GP appointments or *vice versa*. The appointment dataset used is experimental and does not give a complete picture of practice workload. While this study did control for practice IT system, it was not possible to control for other factors that affect which and how appointments are recorded. Only four measures of patient experience from the GPPS were used. These were selected because of the relevance for policymakers and those designing primary care systems. Although these questions serve a purpose, continuity, access, and health needs are complex constructs, the experience of which cannot be fully ascertained by the use of a single question.^24^^,^^25^ There is correlation between some of these variables (for example, access and overall experience) and their relationships with one another are not considered here.

### Comparison with existing literature

Patient satisfaction with access to general practice is often considered to be dependent on how long it takes to get an appointment.^26^ However, research shows that satisfaction with access is dependent on a number of different factors.^25^^,^^27^^,^^28^ It is perhaps not surprising that a greater volume of appointments was positively associated with all patient measures. Perhaps less intuitive was the negative association of same-day and up to 24-hour appointment wait times with patient satisfaction with access and with overall satisfaction. This suggests that patients’ concept of good access to general practice encompasses more than just getting a quick appointment. The dataset does not show the wait or difficulty patients had in making appointments and it is possible that this dissatisfaction may be because practices with larger amounts of on-the-day appointments have worse booking experiences for patients.

The mode of appointments and professional consulted is also linked to patient satisfaction, with the proportion of face-to-face appointments and the proportion of appointments with a GP being linked to all the patient experience measures. There is mixed evidence regarding the acceptability of remote consultations.^29^^,^^30^ Although it appears that certain patients find it useful in particular circumstances, the results of the current study suggest that, at a whole population level, there may be a preference for face-to-face consultations. There is a growing body of research examining the impact of non-medical professionals in primary care.^31^^–^^33^ Previous papers have examined the relationship between funding, number of full-time equivalent GPs and other healthcare professionals and patient experience of access, as recorded through the GPPS.^7^^,^^13^ The findings from the current study are in line with their results and demonstrate it is the volume of GP appointments (not just the number of GPs employed by the practice) that are likely driving positive patient experience.

Taken as a whole, the results paint a nuanced picture of patient satisfaction with primary care and access, and it supports recent qualitative work with patients where they describe access as a concept of ‘human fit’ — encompassing the right type of appointment for the patient with the right clinician at the right time — rather than simply getting a speedy appointment.^25^

### Implications for research and practice

The most significant correlation between all the examined measures of patient experience and satisfaction was the proportion of GP face-to-face appointments. If the effects shown on patient satisfaction in this paper were causal, increasing effect on patient satisfaction were causal, increasing the proportion of GP appointments that are face to face by 1% would result in an increase in patient satisfaction with access similar to adding 10 additional appointments per 1000 patients per month.

The results show that more GP appointments per capita were independently associated with better continuity. Given that the GPPS measures the ability for patients to consult with their preferred GP, this is not surprising. However, this finding varied by appointment modality. Increased proportions of GP face-to-face appointments was strongly associated with improved continuity, whereas an increased proportion of GP telephone appointments was negatively associated with continuity. Continuity of care is a complex concept and the GPPS outcome measure that was examined only provides insight into one aspect of it.^24^ However, given the current NHS plans to improve continuity, and the recognition that continuity is linked to multiple beneficial outcomes, this finding needs further research.^34^^–^^36^

Although telephone and IT-assisted appointments may have an important role to play in general practice, the authors of the current study would cautiously welcome an overall increase in the return to face-to-face consultations. Until recently, simply employing more GPs was not seen as feasible.^37^ However, given that currently there are GPs struggling to find work, this may now be a potential option.^38^^,^^39^ The authors would also welcome measures that free up GP time to enable more patient appointments.

This work used practice-level patient satisfaction data. Future work should establish whether patient experience and its relationship to appointments varies by individual patient characteristics. The link between appointment modality (that is, face to face or remote) needs to be better understood and outcome data other than patient-reported data need to be examined. Work to understand why practices with a greater proportion of on-the-day appointments have poorer patient satisfaction needs to take place. Measuring unmet primary care need and waiting time to book a GP appointment needs to be considered. Future NHS plans envisaged an expanded role in primary care for nursing staff and other non-medical healthcare professionals.^34^^,^^40^ There needs to be research to understand why patients are less satisfied and have a poorer experience in practices with higher proportions of non-GP appointments.

## Data

The data used in the study are publicly available through the NHS Digital and GP Patient Survey websites.
